# Robotik und computergestützte Chirurgie

**DOI:** 10.1007/s00104-023-01822-z

**Published:** 2023-03-27

**Authors:** Andreas Seekamp

**Affiliations:** Klinik für Orthopädie und Unfallchirurgie, Universitätsklinikum Schleswig-Holstein, Campus Kiel, Arnold-Heller Straße 3, Haus C, 24105 Kiel, Deutschland

Wie die Medizin insgesamt unterliegt auch die Chirurgie einem fortwährenden Wandel, der in erster Linie durch den technischen Fortschritt getrieben ist, begleitet von dem Bestreben, das Spektrum chirurgischer Maßnahmen zu erweitern, die Sicherheit des Patienten zu erhöhen und die chirurgischen Zugänge zu minimieren.

Wesentliche Schritte in der jüngeren Geschichte der Chirurgie waren diesbezüglich die Antisepsis, die Verwendung der Röntgenstrahlung und die Etablierung der Anästhesie. Der heutige ebenfalls als revolutionär zu betrachtende Fortschritt besteht darin, die intraoperative Visualisierung des Situs um die dritte Ebene bis hin zur dreidimensionalen Darstellung zu etablieren und die manuellen Fertigkeiten des Operateurs durch Miniaturtechnik zu verfeinern und zu präzisieren. Es ist bereits 40 Jahre her, dass anhand von Fallbeispielen erste Techniken rechner- bzw. computerunterstützter Operationen vorgestellt wurden [1]. Mittlerweile haben die unterschiedlichsten Techniken in nahezu allen Disziplinen der Chirurgie Einzug gehalten [2, 3] und es kristallisiert sich mittlerweile heraus, in welchen Bereichen und bei welchen Operationen die Robotik und computerunterstützte Chirurgie tatsächlich sinnvoll und nachhaltig und für den Patienten von wirklichem Nutzen sind.

Heute lassen sich verschiedene Systeme voneinander abgrenzen, sodass zunächst eine Begriffsbestimmung erforderlich erscheint, welche auch in den nachfolgenden Beiträgen aufgegriffen wird. Bereits in den 1990er-Jahren wurden Operationsroboter entwickelt, die im Rahmen der Endoprothetik an Hüft- und Kniegelenken den Knochen vorfrästen, um die entsprechenden Implantate passgenau einbringen zu können. Der technische Aufwand für diese Operationen war jedoch sehr hoch. Es waren zusätzliche Röntgenaufnahmen erforderlich und die Operationszeiten verlängerten sich erheblich. Postoperativ zeigte sich eine erhöhte Komplikationsrate mit frühen Wundinfekten und im Verlauf gelockerten Hüftprothesen. Die Rolle des Operateurs war bei diesem Verfahren eher passiv, nachdem sämtliche Einstellungen für den Fräsvorgang für den Roboter eingestellt waren. Nachhaltig durchsetzen konnte sich dieses System letzten Endes nicht [4, 5].

Ebenfalls in den 1990er-Jahren nahm die Entwicklung computer- bzw. rechnergestützter Operationen ihren Beginn. Ziel war es, insbesondere im Bereich der Becken- und Wirbelsäulenchirurgie die dritte axiale Ebene darzustellen, die sich mit den konventionellen Bildwandlereinstellungen nicht abbilden lässt. Hierdurch sollte das Setzen von Implantaten, wie z. B. Pedikelschrauben an der Wirbelsäule, sicherer gestaltet werden können. Das Prinzip beruht auf einer dreidimensionalen Erfassung der intraoperativ verwendeten Instrumente über eine 3‑D-Kamera, deren Datensatz mit einer zuvor durchgeführten computertomographischen Untersuchung des Patienten intraoperativ abgeglichen wird. Die Verfahrenstechnik war zu Beginn sehr aufwendig, dennoch wurden vielerlei Anwendungsmöglichkeiten für verschiedene Operationen am Bewegungsapparat erprobt. Nachdem die Techniken verfeinert waren, hat sich dieses System der computer- bzw. rechnergestützten Operationstechnik nachhaltig in der Wirbelsäulen- und Beckenchirurgie durchsetzen können [6, 7]. Die Kollegen *F. Zimmermann *und* J. Franke* beleuchten den aktuellen Stand der Entwicklung dieser Techniken in Orthopädie und Unfallchirurgie. Explizit für die Wirbelsäule werden die aktuellen Möglichkeiten computergestützter Verfahren von *T. Picht *und* P. Vaikoczy* erläutert.

Aktuell konzentriert sich die Entwicklung in diesem Bereich darauf, die dreidimensionale Erfassung der Instrumente direkt mit der intraoperativen Bildgebung zu verkoppeln und damit zum einen den Geräteaufwand zu reduzieren und zum anderen den Abgleich, das sog. „matching“, der unterschiedlich generierten Bilder zu verbessern. Dies kann gelingen, indem man die Erfassung der Operationsinstrumente über ein Kamerasystem vornimmt, welches in den Bildverstärker bzw. Bildwandler, der auch intraoperativ eine dreidimensionale Bildgebung ermöglicht, integriert ist [8]. Erste Erfahrungen mit dieser Technik werden von *M. Wegner *und* S. Lippross* in ihrem Beitrag dargestellt. Hierdurch wird es grundsätzlich möglich, eine virtuelle Realität zu schaffen, in welcher sodann die Implantate gesetzt werden, ohne dass unter den chirurgischen Maßnahmen wiederholt Röntgenaufnahmen angefertigt werden müssen. Aufgrund der Tatsache, dass durch diese Technik ein Zielkorridor vorgegeben wird, in welchem z. B. in der Wirbelsäulenchirurgie Pedikelschrauben zu setzen sind, hat man eine Ähnlichkeit mit dem Leitsystem der Verkehrsluftfahrt gesehen und spricht daher auch von „navigiertem“ Operieren bzw. von Navigationssystemen in der Chirurgie.

In den letzten Jahren hat diese Art der computerunterstützten Operationstechnik auch wieder Einzug in die Hüft- und Kniegelenksendoprothetik gefunden. Im Vergleich zu der vorangegangenen und wieder verlassenen voll automatisierten Robotik im Bereich der Endoprothetik werden jetzt lediglich die Säge- und Fräsvorgänge rechnergestützt vorgenommen [9]. Die Instrumente selbst bleiben komplett in der Hand des Operateurs, der nunmehr in die Lage versetzt wird, quasi in einem aktuell generierten dreidimensionalen Modell die Sägeschnitte und Fräsvorgänge für die zu implantierende Prothese vorzunehmen [10]. Im Bereich der Orthopädie und Unfallchirurgie wurde die initial angedachte Robotik in den letzten Jahren komplett durch computerunterstützte Operationstechniken abgelöst. Aktuell wird aber auch die Automatisierung wieder aufgegriffen [11], wobei der Roboterarm heute mit mehr patientenindividuellem Datenmaterial computergestützt gesteuert wird und ihm so quasi auch eine gewisse „Haptik“ vermittelt wird, soll heißen der Roboterarm kann auf sensible Strukturen reagieren und diese umfahren. Diese spannende und zukunftsweisende Technik wird in dem Beitrag von *J. Reinhold *und* S. Lippross* näher beschrieben.

In der viszeralen Chirurgie, der Gynäkologie und der Urologie hat sich hingegen eine Fortentwicklung aus der endoskopischen Chirurgie ergeben, die heute als roboterunterstützte Chirurgie bezeichnet werden kann. Ziel dieser Entwicklung war es ursprünglich, in einem übersichtlichen Operationsfeld, welches endoskopisch gut darzustellen ist, die Wendigkeit und Eingriffsmöglichkeit des Operateurs zu verbessern. Hierzu war es erforderlich, dass die Instrumente nicht mehr direkt von dem Operateur geführt werden, sondern seine operativen Schritte über Computerarme, die endoskopisch eingebracht werden, im Situs in einem Verkleinerungs- oder Vergrößerungsmodus umgesetzt werden. Dieses System hat sich zunächst in der Urologie rasch etabliert und hier insbesondere bei der Prostataektomie. Diese Operation schien sehr geeignet zu sein, da das Operationsfeld übersichtlich war und keine wiederholten Neueinstellungen des Situs vorgenommen werden mussten [12]. Der Operateur kann nun, selbst an einer Konsole sitzend, die Operation ohne größere Assistenz durchführen, indem seine Handlungen direkt vor Ort bis hin zu mikrochirurgischen Techniken von den robotergeführten Instrumentenarmen durchgeführt werden. Anpassungen und Erweiterungen dieses Systems ermöglichen es heute, dass auch größere viszeralchirurgische Eingriffe [13] und gynäkologische Eingriffe mit diesem System durchgeführt werden. Die vielseitige Anwendbarkeit dieser Technik in der Viszeralchirurgie wird von den Kollegen *J. Umstadt *und* M. Kallenberger* dargestellt.

Im eigenen Vorgehen haben wir diese Technik auch an Eingriffen der thorakalen und lumbalen Wirbelsäule für eine ventrale Stabilisierung erprobt. Letzten Endes sind aber die bisherigen Instrumente nicht dafür geeignet, einen Bandscheibenraum auszuräumen oder aber ein knöchernes Fenster zu schaffen, um hier ein wirbelkörperersetzendes Implantat einzubringen [14]. Die hierfür notwendige Kraftanwendung bzw. Kraftübertragung sind aktuell von keinem der zur Verfügung stehenden Systeme zu leisten. Dennoch bleibt der Vorteil, minimal-invasiv einen Hohlraum zu schaffen, z. B. im Bereich der lumbalen Lendenwirbelsäule, in welchem sodann endoskopisch und roboterunterstützt zumindest der ventrale operative Zugang bis zur Wirbelsäule präpariert werden kann. Auch für den Zugang zur thorakalen Wirbelsäule kann dieses System von Nutzen sein, wobei der apparative Aufwand zur Installation dieses Systems, um es nur für den chirurgischen Zugang zur thorakalen Wirbelsäule zu nutzen, etwas aufwendig erscheint. Für operative Eingriffe an der thorakalen Wirbelsäule von ventral ist daher aktuell der reinen endoskopischen Technik der Vorzug zu geben.

Roboterunterstützte Chirurgie verfügt über eine vielseitige Anwendbarkeit in der Viszeralchirurgie

Neben der roboterunterstützten Chirurgie für Körperhöhlen wurde vor kurzem auch ein etwas weniger großdimensioniertes System entwickelt, welches für mikrochirurgische Techniken im Oberflächenbereich geeignet ist bzw. in Bereichen außerhalb vorgegebener oder vorzuformender Körperhöhlen. Vergleichbar mit dem großen Robotiksystem wird auch bei dem kleineren System mit Roboterarmen gearbeitet, wobei nunmehr nicht endoskopische Verfahren im Vordergrund stehen sondern die Roboterarme mikrochirurgische Techniken direkt an Oberflächen durchführen können. Auch hier sitzt der Operateur an einer kleinen Konsole und führt die chirurgischen Maßnahmen mit zwei frei beweglichen „joy sticks“ durch. Der operative Situs wird über ein Operationsmikroskop dargestellt und seitens des Operateurs und ggf. eines Assistenten eingesehen. Geeignet ist dieses System für die Handchirurgie, die mikrochirurgische und plastische Chirurgie sowie für die Tumorchirurgie auf dem Hals-Nasen-Ohren-ärztlichen und Mund-Kiefer-Gesichts-chirurgischen Gebiet. Weitere Details sind dem Beitrag von den Kollegen *L. Grünherz *und* E. Gousopoulos* zu entnehmen, in dem der Nutzen dieser Technik in der plastischen Chirurgie betrachtet wird.

Bei allen auf Robotik basierenden Systemen wird ein dreidimensionales Bild des Operationsfeldes erstellt

Allen Systemen, die auf einer Robotik oder Rechnerunterstützung basieren, ist gemeinsam, dass ein dreidimensionales Bild des Operationsfeldes erstellt wird. Insofern ist es naheliegend, derartig generiertes Material auch für Trainingszwecke bis hin zur Simulation zu verwenden. *M. Blätzinger* stellt in seinem Beitrag zukünftige Möglichkeiten der Simulation in der Chirurgie dar. Es versteht sich von selbst, dass die Lernkurve dieser Anwendungen sehr flach ist und man daher zukünftigen Operateuren in der Ausbildung die Simulation solcher Operationen ermöglichen muss [15]. All diese Dinge befinden sich bereits in Etablierung, sodass unter Verwendung von 3‑D-Brillen derartige Operationen am Simulator durchgeführt werden können, so wie man dies schon seit Jahren von Computerspielen kennen mag. Für die Zukunft ist zu erwarten, dass die Techniken noch weiter verfeinert werden und dadurch die Präzision noch weiter zunimmt.

Eine Herausforderung wird noch die intraoperative Bildgebung bleiben. Es ist zu erwarten, dass intraoperativ gefährdete und zu umgehende Zonen in der dreidimensionalen Darstellung, ähnlich wie in einem anatomischen Lehrbuch, besonders hervorgehoben sein werden, um eine intraoperative Verletzung gefährdeter Strukturen zu vermeiden. Ebenfalls ist absehbar, dass im Rahmen der Bildgebung unter Verwendung bestimmter Marker z. B. ein zu resezierender Tumor bildlich dargestellt werden kann und somit die Resektion, z. B. intrahepatischer oder intrazerebraler Tumoren, präziser und schonender gelingen kann.

In den folgenden Beiträgen werden die hier nur kurz dargestellten Techniken detailliert erläutert, ihre technische Umsetzung dargestellt und Vor- und Nachteile diskutiert. In der Summe bleibt festzustellen, dass die Chirurgie der Zukunft zunehmend technikbasiert sein wird. Ziel dieser Entwicklung muss es weiterhin sein, die chirurgische Präzision zu erhöhen und damit zum Wohl und der Sicherheit der Patienten beizutragen.
